# Properties of Polyvinyl Alcohol Films Composited With Hemicellulose and Nanocellulose Extracted From *Artemisia selengensis* Straw

**DOI:** 10.3389/fbioe.2020.00980

**Published:** 2020-08-25

**Authors:** Sheng Xu, Mingjun Jiang, Qiuhao Lu, Siyuan Gao, Jiao Feng, Xin Wang, Xun He, Kequan Chen, Yan Li, Pingkai Ouyang

**Affiliations:** State Key Laboratory of Materials-Oriented Chemical Engineering, College of Biotechnology and Pharmaceutical Engineering, Nanjing Tech University, Nanjing, China

**Keywords:** agricultural wastes, *Artemisia selengensis* straw, hemicellulose, cellulose nanocrystals, biodegradable composite films, mechanical properties

## Abstract

*Artemisia selengensis* straw is an agricultural residue with great potential as a renewable resource because it is rich in lignocellulose. In this study, *A. selengensis* straw was used as a source of hemicelluloses (ASH) and cellulose nanocrystals (ASCNC) to produce biodegradable films. Different content levels of ASCNC were used as additives to improve composite films with ASH and polyvinyl alcohol (PVA). Various mechanical and hydrophobic properties of the films were analyzed. The composite films enhanced by ASCNC exhibited greater strength and were more effective as a barrier to water vapor when compared to that of the control ASH/PVA film. The tensile strength of the composite film was increased 80.1% to 36.21 MPa with ASCNC loading exceeding 9%, and the water vapor transmission rate decreased 15.45% when 12% ASCNC was added. Furthermore, the ASCNC-enhanced ASH/PVA composite film reduced a greater amount of light transmission than the control film.

## Introduction

China produces abundant amounts of agricultural and forestry wastes annually, equivalent to approximately 1.2 billion tons of standard coal equivalent as estimated to 2020 (Chen and Shi, 2017). With decreasing reserves of petrochemical resources and increasing pressure for environmental protection, researchers have gradually turned their attention to these large sources of waste as potential renewable resources (Ho et al., 2014; Trninic et al., 2016; Jia et al., 2018). Straw, from crops such as wheat, rice, and corn, is a type of agricultural residue abundant in lignocellulose, which contains cellulose, hemicellulose, and lignin (Sindhu et al., 2016). The availability and bioenergy potentials of the current biomass feedstocks have caused great concern on efficiently producing biofuels involving bioethanol and biogas from such agricultural residues (Wang Y. et al., 2016; Zhao et al., 2016; Alam et al., 2019; Gao et al., 2019). However, there is still a growing demand to develop new applications of straw organic matter, for the benefits of improving agricultural productivity, reducing waste, and protecting the natural environment.

Previous studies have reported that the hemicellulose in lignocellulose can be used in production of biodegradable composite films with polyvinyl alcohol (PVA). The composite films with low oxygen permeability could be used for food packaging material; however, the applications of hemicellulose film are limited because of its high hydrophilicity and the resulting poor mechanical properties (Hansen and Plackett, 2008). Film hydrophobicity could be enhanced by optimizing alkali-catalyzed organosolv treatment conditions before hemicellulose extraction (Mugwagwa and Chimphango, 2020). Incorporation of additives, such as plasticizers, reinforcing agents, and modifiers, into the production of hemicellulose film can increase the mechanical strength and heat resistance and reduce the hydrophilicity of this type of film (Cacciotti et al., 2014; Wang et al., 2014; Pereira et al., 2017). Cellulose nanocrystals (CNC) are derived from cellulose through a combination of mechanical, chemical, and enzyme treatments and have a high specific surface area, high reactivity, high Young’s modulus, excellent mechanical properties, and good biocompatibility and biodegradability (George and Sabapathi, 2015; Kargarzadeh et al., 2018). One important utilization of CNC for various applications is to act as a reinforcing agent in polymer (nano)composites (Lee et al., 2016; Huang et al., 2018; Shuai et al., 2020). In the production of gelatin-CNC bionanocomposite films by continuous casting, adding only 0.5 wt% of CNC resulted in an increase of 77 and 48% in the tensile strength and Young’s modulus of gelatin, respectively (Leite et al., 2020). At 40 wt% rod-like CNC loading, the strength and stiffness of CNC/PVA composite fibers reached 880 MPa and 29.9 GPa, respectively, exceeding the properties of most other nanocellulose-based composite fibers (Lee et al., 2016).

*Artemisia selengensis* is widely planted as a cash crop in southern China, with an annual output of 5 × 10^7^ kg of saleable crop material accompanied by a straw output of ∼4 × 10^7^ kg in Nanjing, the capital city of Jiangsu province (Xie et al., 2014; Zhang, 2019). It contains flavonoids, vitamins, alkaloids, and other valuable ingredients and is rich in lignocellulosic components (Peng et al., 2011; Wang J. et al., 2016). Several studies have reported on extracting compounds with antioxidant activity (Zhang et al., 2014; Wang J. et al., 2016) or gas production from *A. selengensis* straw (Xie et al., 2014). However, the isolation and utilization of lignocellulose components in *A. selengensis* straw have received little attention. In the present study, hemicelluloses (ASH) and cellulose nanocrystals (ASCNC) were separately extracted from *A. selengensis* straw, and a new composite film made of ASCNC, ASH, and PVA materials was subsequently developed, where the ASCNC obtained from the cellulose in *A. selengensis* straw was added as a reinforcing agent to a PVA and ASH matrix composite film. The effects of ASCNC content on the mechanical properties, hydrophobic properties, and light transmission of the composite film were studied. The composition and microstructure of the composite film were investigated by Fourier transform infrared spectroscopy (FTIR) and scanning electron microscopy (SEM).

## Materials and Methods

### Separation of Lignocellulose Components From *A. selengensis* Straw

*Artemisia selengensis* straw collected from Baguazhou in Nanjing was used as the raw material. The straw was milled to 0.5 mm in a centrifugal grinding mill QM-3SP2-CL (Nanjing NanDa Instrument Plant, Nanjing, China) at 500 rpm for 2 h and passed through a 32-mesh sieve. Pigments, waxes, and other impurities in the straw were first removed using a Soxhlet extractor with toluene/alcohol solution (2:1, v/v) at 150°C for 6 h. The dewaxed *A. selengensis* straw powder was treated by microwave (750W) assisted alkali/alcohol solution (1% NaoH, w/v and 70% alcohol, w/v) at 90°C for 1.5 h, washed with water five times, and filtered to extract the lignin. Filter residue (100 g) was treated by 4% sodium hydroxide solution (2 L) at 70°C for 1.5 h then filtered and precipitated by alcohol to obtain ASH. The secondary filter residue (10 g) was treated twice by sodium 1% chlorite solution (200 mL) at 75°C for 1 h, filtered, then washed with water five times and dried to obtain *A. selengensis* straw cellulose (ASC). These conditions were modified from those optimized in previous work (Chen et al., 2019). All chemicals used in this study were purchased from Sinopharm Chemical Reagent Co., Ltd (Shanghai, China).

### Preparation of ASCNC

The ASCNC was extracted from the ASC by acid hydrolysis, as described by Nascimento et al. (2014) with modifications. The ASC (5 g) was mixed in a flask with 100 ml of sulfuric acid solution (50%, w/v) in a 55°C water bath and stirred for 1.5 h before 500 ml of water was added. The mixture was centrifuged for 15 min, then 1 L of deionized water was added, and the mixture was centrifuged again until the supernatant became a white suspension of ASCNC. Subsequently, the suspension was dialysis-treated for 72 h to remove H^+^. The suspension was poured into a glass plate and then freeze-dried at −60°C overnight using a vacuum freeze dryer (LGJ-10D, Beijing Sihuan Qihang Technology Co., Ltd., Beijing, China) to obtain a solid sample.

The chemical composition of *A. selengensis* straw was determined according to the following methods: cellulose and hemicellulose (TAPPI T257 om-09); lignin (TAPPI T222 om-11). The mass yields (%) were calculated as the ratios of the extracted (g) ASH, ASC, and lignin to the corresponding substances in straw (g). The purity of the extracted components refers to the percentage of each in the obtained sample.

### Preparation of ASCNC-Enhanced ASH/PVA Film

Polyvinyl alcohol (1.5 g, polymerization 1750 ± 50, alcoholysis of 99%, Sinopharm Chemical Reagent Co., Ltd., Shanghai, China) and 0.5 g water-soluble hemicellulose were mixed with ASCNC suspension (Wang et al., 2014), and the concentration of ASCNC in the mixture was controlled at 0, 3, 6, 9, and 12% (w/w), respectively. The five concentrations of suspensions were heated at 90°C while stirring for 90 min, then sonicated at 150 w, 40 Hz, using an ultrasonic cleaner (SB-5200DTD, Ningbo Scientz Biotechnology Co., Ltd., Zhenjiang, China) for 10 min to remove bubbles. Subsequently, the mixtures (15 mL) were cast in different Petri dishes with a diameter of 9 cm and dried at 50°C for 2 h. The dried films were stored in a humidity chamber at a constant temperature (25°C) and 50% relative humidity for at least 2 days before subsequent tests.

### Scanning Electron Microscopy and Transmission Electron Microscopy

The surface and hand-broken cross sections of the films and ASCNC were observed using a scanning electron microscope (SEM) (Zeiss, Oberkochen, Germany) with an acceleration voltage of 3 kV, after gold-coating (electric current: 10∼20 A, 90 s). The diameter, length, and aspect ratio of ASCNC was determined using a transmission electron microscope (TEM) (JEOL, JEM 2100, Japan). The ASCNC suspension (0.01%, w/v) was absorbed on a copper mesh then placed into the TEM instrument for observation. The diameter and length were measured using the image processing software ImageJ, and average values of 50 measurements were presented.

### Optical Transmittance and Water Vapor Permeability

The film was cut into strips of 40 mm × 10 mm and pasted on the walls of a quartz cuvette to measure light transmittance through the film at different wavelengths (400–800 nm) with a spectrophotometer (UV-1200, Aoyi Instruments Shanghai Co., Ltd., Shanghai, China). Five grams of anhydrous calcium chloride was added to each of the five identical centrifuge tubes and then sealed by the prepared film material. The sealed tubes were held in a humidity chamber at a constant 25°C and 75% humidity and weighed every 24 h. The water vapor transmission rate (WVTR) and the water vapor permeability (WVP) of the film were calculated using the following formula:

$$\text{WVTR}=\frac{\Delta \,\text{m}}{\text{At}}$$

$$\text{WVP}=\frac{{\mathrm{WVTR}}^{*}\,L}{\Delta \,\text{p}}$$

where Δm is the change in mass of the centrifuge tube; A is the inner area of the orifice of the centrifuge tube; t is time; L is the thickness of the film; Δp is the partial water vapor pressure difference (Pa) across the two sides of the film.

### Fourier Transform Infrared (FTIR)

The spectra of samples (PVA, ASH, ASCNC, and films) were obtained using an infrared spectrometer (Nicolet iS5, Thermo Scientific, United States) at a resolution of 0.5 cm^–1^ with 16 scans at a range of 4,000–500 cm^–1^.

## Results and Discussion

### Separation of *A. selengensis* Straw Lignocellulose

Previous research reported that the compositions of cellulose, hemicellulose, and lignin in *A. selengensis* straw were 35.6, 25.6, and 26.5 wt%, respectively (Chen et al., 2019), comparable with straws from other crops like wheat and maize, in terms of major wall polymer compositions (Wang Y. et al., 2016). A microwave-assisted organic solvent method was used to obtain lignin. Alkali solution was used to dissolve and recover the hemicellulose. Acidic sodium chlorite solution was used to purify the cellulose. The yields of lignin, hemicellulose, and cellulose were 59.3, 73.0, and 80.6%, respectively. After two treatments with sodium chlorite solution, the cellulose purity was as high as 93.8%.

### Preparation and Characterization of ASCNC

#### Preparation of ASCNC

In the process of sulfuric acid hydrolysis, sulfuric acid can effectively degrade the amorphous region in the molecular structure of cellulose at a suitable temperature, but also partly hydrolyze crystalline zones of the cellulose, resulting in a shortening of CNC whisker length (Rosa et al., 2010). Therefore, it is necessary to examine the effects of different conditions (acid concentration, hydrolyzed temperature, and reaction time) on the yield of ASCNC.

**FIGURE 1:**
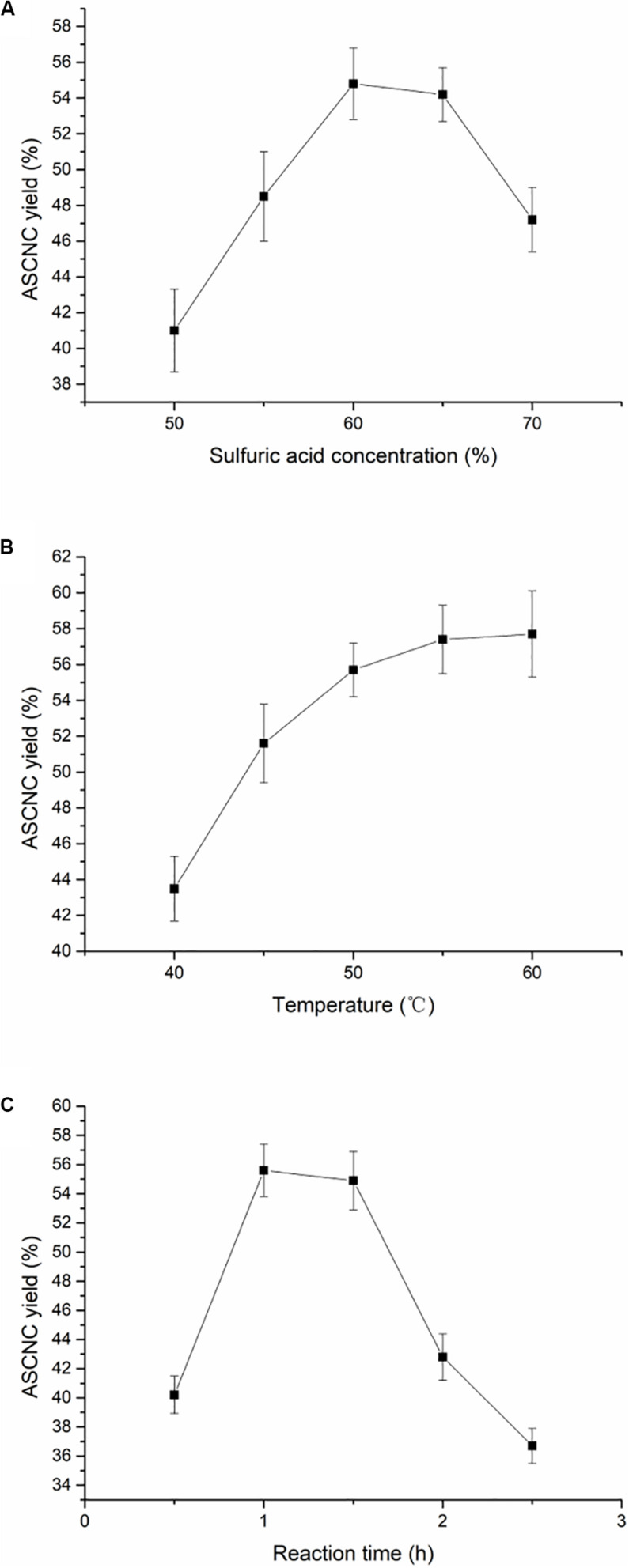
Effect of reaction conditions on the yield of ASCNC. **(A)** Sulfuric acid concentration; **(B)** reaction temperature; **(C)** reaction time.

The yield of ASCNC was relatively high when the mass fraction of sulfuric acid was between 60 and 65% (Figure 1A). When the concentration of sulfuric acid was low, the degree of acid hydrolysis was far from enough to break hydrogen bonds, glycosidic bonds, and non-crystalline regions in the cellulose molecule. A large amount of cellulose in the crystalline region failed to be released. On the other hand, at an acid concentration higher than 65%, the monosaccharides and disaccharides were detected in the hydrolysate, which may be caused by excessive hydrolysis of cellulose. Furthermore, a high concentration of sulfuric acid means that carbonization causes a decrease in CNC yield (Kim et al., 2001). Similarly, when the temperature was relatively low, it was not conducive to the acid hydrolysis reaction. Excessively high temperatures can also cause carbonization and degradation reactions (Figure 1B). As the reaction time increased, cellulose degraded into monosaccharides; the disaccharide reaction and the hydrolysis of the amorphous region occurred simultaneously. After 1.5 h, the amorphous region was substantially completely hydrolyzed, cellulose was extensively degraded into glucose and other substances, and the ASCNC yield sharply decreased (Figure 1C). Finally, the ASCNC yield was 54.89% under the optimized reaction conditions (60% acid concentration, 55°C and 1.5 h).

#### Morphological Analysis of ASCNC

**FIGURE 2:**
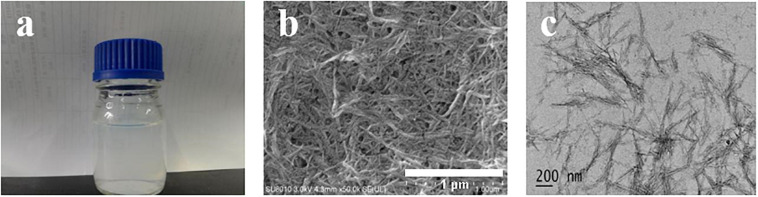
Image of ASCNC. **(a)** ASCNC suspension; **(b)** SEM image of ASCNC; **(c)** TEM image of ASCNC.

As shown in Figure 2a, the suspension of ASCNC was white yet transparent. After freeze-drying the suspension, a white silky solid was obtained. SEM images (Figure 2b) showed that during acid hydrolysis of ASCNC, nanocrystalline cellulose whiskers were successfully produced. The diameter of nanocrystalline cellulose was nanometers in size, and the structure was in the form of a long rod. TEM can be used to observe the microscopic morphology of a single ASCNC (Figure 2c). A nanocrystal has a diameter of about 10 nm and a length of about 200 nm. Many nanofibers agglomerated together due to the hydrogen bonds.

**FIGURE 3:**
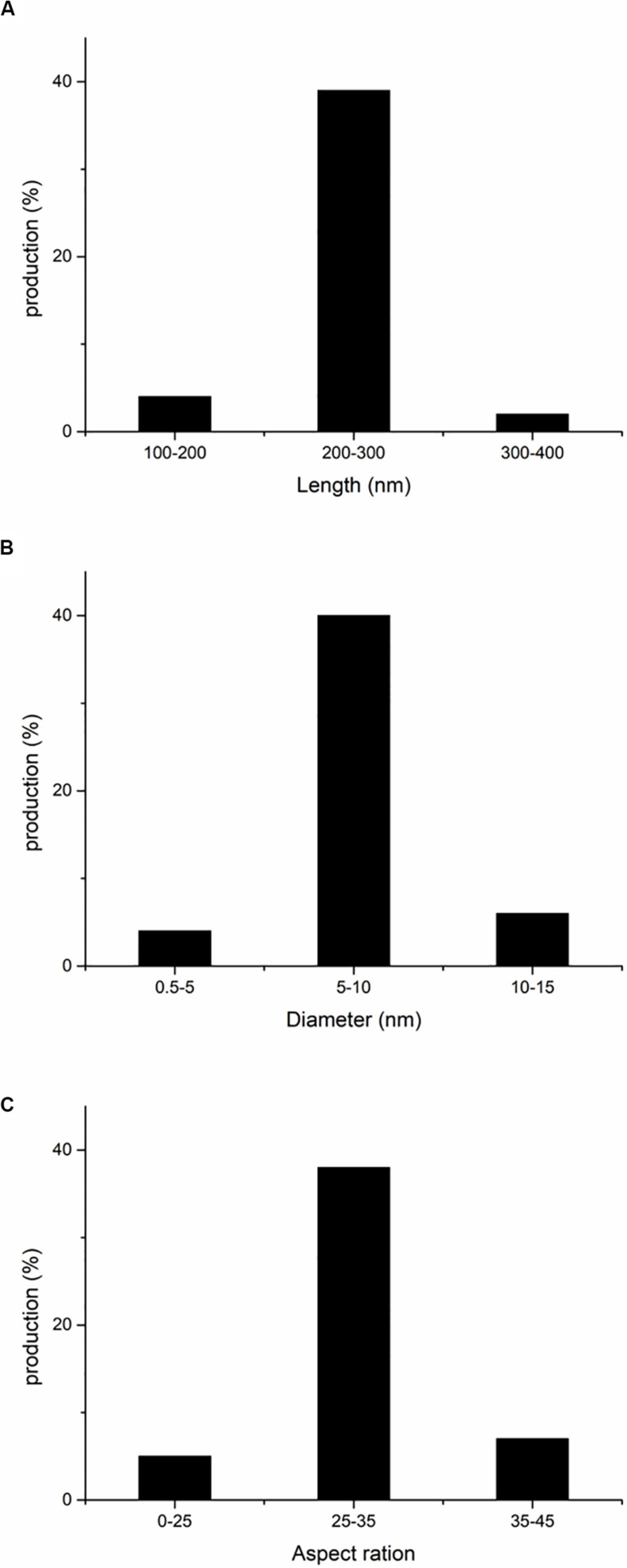
Distributions of morphological data of ASCNC. **(A)** Length; **(B)** diameter; **(C)** aspect ratio.

Using TEM, we selected 50 ASCNC whiskers produced under the optimized conditions. The fiber diameter, fiber length, and length-to-diameter ratio were used as indices to observe the distribution of the three morphological measures, which are shown in Figure 3. The size distributions of ASCNC mainly concentrated at 200–300 nm in length and 5–10 nm in diameter. The similarity of the morphology among nanocrystals ensures the homogeneity and stability of their properties. Studies have shown that CNC, with a high length-to-diameter ratio that is expected to give a good reinforcing effect, may play an important role in improving the physical properties of composite materials (Rhim et al., 2015). In this study, most of the aspect ratios of ASCNC were at the range of 25–35, which was comparable to CNC from barley, pea hull, and *Agave tequilana* (Chen et al., 2009; Espino et al., 2014).

### Characterization of ASCNC-Enhanced ASH/PVA Composite Film

#### Mechanical Properties of Composite Films

**FIGURE 4:**
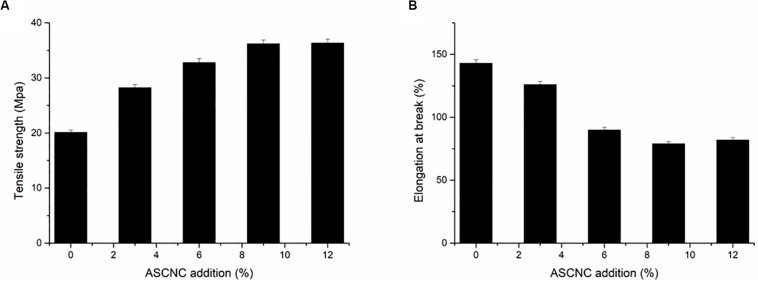
Effect of ASCNC addition on mechanical properties of films. **(A)** Tensile strength; **(B)** elongation at break.

ASCNC was added as a reinforcing agent to a composite film of PVA and ASH matrix. The effect of the addition of various concentrations of ASCNC (0 to 12%) on the thickness and moisture content of composite films was determined (Table 1). The film thickness increased, and the moisture uptake decreased as the ASCNC content increased in the nanocomposite films, which was probably led by the water insolubility and high crystallinity of CNC (Alam et al., 2019). Mechanical properties are the most important performance indicators for packaging materials. Addition of ASCNC can effectively improve the mechanical properties of composite films (Figure 4A). The tensile strength of the film increased by 50% upon adding 3% ASCNC. When 9% ASCNC was added, the tensile strength of the film reached an optimal level of 36.21 MPa, which was 80.1% stronger than the film without ASCNC. The increased strength is likely due to the rod-like nanostructures of ASCNC that promote a uniform dispersion of nanocrystals in the matrix and the strong hydrogen bonding property of CNC that created a tendency to aggregate (Fortunati et al., 2013). In contrast to results of the tensile strength, the elongation at break of the composite films decreased from 143 to 79% with increasing amounts of ASCNC up to 9% (Figure 4B), which was a result of physical entanglement and hydrogen bonding between CNC and the matrix. Since the CNCs are not as deformable as the matrix, the strong interactions between the CNCs and matrix molecules do not allow PVA to elongate. The reduction in elongation at break of PVA was also found in nanocomposites based on PVA and cellulose nanofiber (Kakroodi et al., 2014, 2015). Therefore, the formation of a percolating network-like architecture inside the matrix that is responsible for the enhanced mechanical properties of nanocomposite films, which was in accordance with the results obtained from the following infrared test and electron microscopy test.

**TABLE 1:** Basic properties of ASCNC-enhanced ASH/PVA composite films.

ASCNC (wt%)	Film thickness (μm)	Moisture content (%)
0	54 ± 4	9.8 ± 1.4
3	55 ± 4	9.0 ± 0.9
6	57 ± 3	8.9 ± 1.2
9	58 ± 3	8.8 ± 1.1
12	58 ± 4	8.6 ± 1.2

#### Light Transmission of Composite Films

**FIGURE 5:**
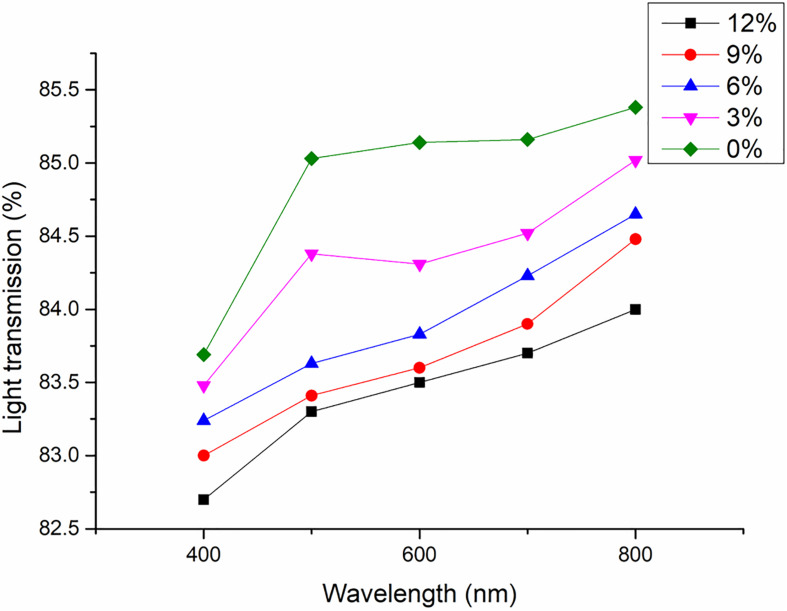
The effect of the addition of ASCNC on the transparency of composite films. Amount of ASCNC: 0% (◆); 3% (▼); 6% (▲); 9% (•); 12% (■).

As transparency is considered an important parameter for packaging and applications, light transmission performance of the composite films was measured at a wavelength from 400 to 800 nm, indicating a slight decrease with increased amounts of ASCNC. Transmittance of the ASH/PVA composite film was 85% at a wavelength of 700 nm. The transparent nature is almost not affected by the addition of ASCNC (3–12%), without a significant reduction (less than 1.5%) in the amount of light being transmitted (Figure 5). This phenomenon is common in composite films enhanced by CNC and is primarily due to the nanoscaled dispersion of CNC within the polymer matrix (Fortunati et al., 2013). Without ASH supply, the transmittance of the ASCNC/PVA composites is probably supposed to be higher as noted in Fortunati et al. (2013).

#### WVP of Composite Films

**FIGURE 6:**
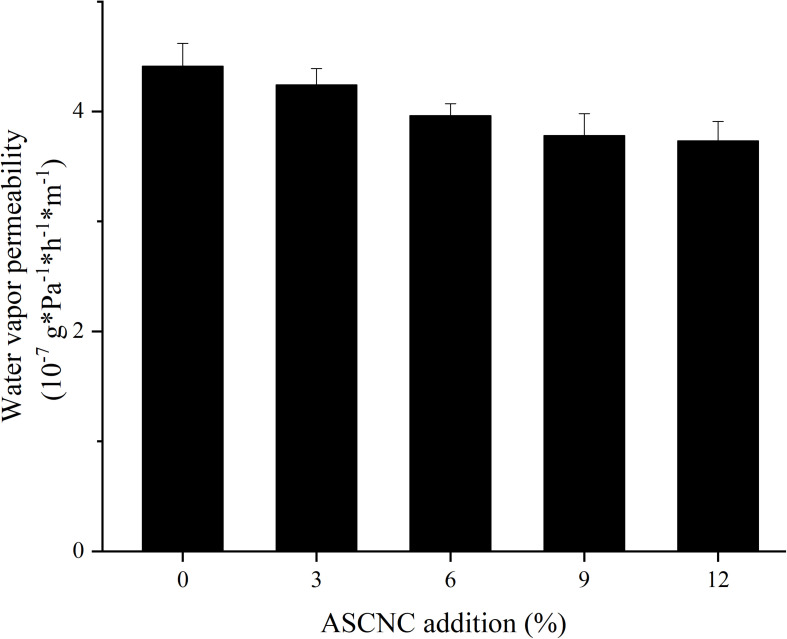
Effect of the amount of ASCNC on the water vapor permeability of film.

The effect of ASCNC on the water-repellent properties of the ASH/PVA film was examined by measuring the water vapor transmission rate and swelling coefficient (Figure 6). The water vapor transmittance rate of the film was improved by the addition of ASCNC. As the content of ASCNC increased, the water vapor transmission rate of the film decreased. The WVP reached a minimum of 3.73^∗^10–7 g/Pa^∗^h^∗^m, which was a composite film with 12% ASCNC added, which was 15.23% lower than that of the control group. This indicates that the addition of ASCNC may inhibit the movement and binding of water molecules between ASH and PVA molecules (Pereira et al., 2017). Empty spaces in the hemicellulose and PVA films were likely filled in by ASCNC, making the structure of the films more compact (Li et al., 2009). On the other hand, the rich hydroxyl groups on the surface of ASCNC, which can form between hemicellulose and PVA to form a structural network of stable hydrogen bonds, may cause a decrease in the sensitivity of film materials to water molecules (Yu et al., 2015).

#### Fourier Transform Infrared Spectroscopy Analysis

**FIGURE 7:**
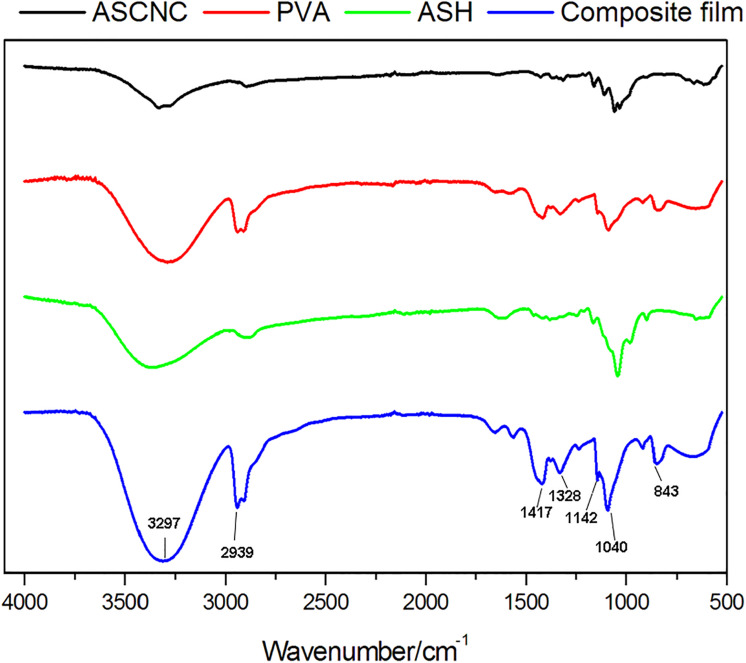
Infrared spectrum of the composite film and its major components.

Fourier transform infrared spectroscopic (FTIR) analysis was performed to examine the chemical structure of the composite film and raw materials. Two main dominant absorption regions appeared in all the FTIR spectra curves of the composite film (Figure 7, blue line). One was from 3297 to 2939 cm^–1^ in the high-wave-number region, and the other one was from 1654 to 843 cm^–1^ in the low-wave-number region. The wide region around 3297 cm^–1^ and peaks at 2939 cm^–1^ and 1089 cm^–1^ in the spectra of all the samples corresponded to stretching vibrations of O–H, C–H, and C–O groups, respectively (Chen et al., 2011; Liu et al., 2016). The peaks at 1417, 1328, 1142, and 843 cm^–1^ for C–H bending, C–H wag-ging, crystalline domains of PVA, and rocking mode, respectively, were the characteristic peaks of PVA (Ahad et al., 2012; Sun et al., 2014). The peak at 1654 cm^–1^ was attributed to the H–O–H stretching vibration of the adsorbed water due to the OH groups (Alemdar and Sain, 2008). The FTIR results indicated that the three substances, PVA, ASH, and ASCNC, were tightly integrated during the preparation of the film and no chemical reactions occurred that formed a new substance.

#### Morphology of Composite Films

**FIGURE 8:**
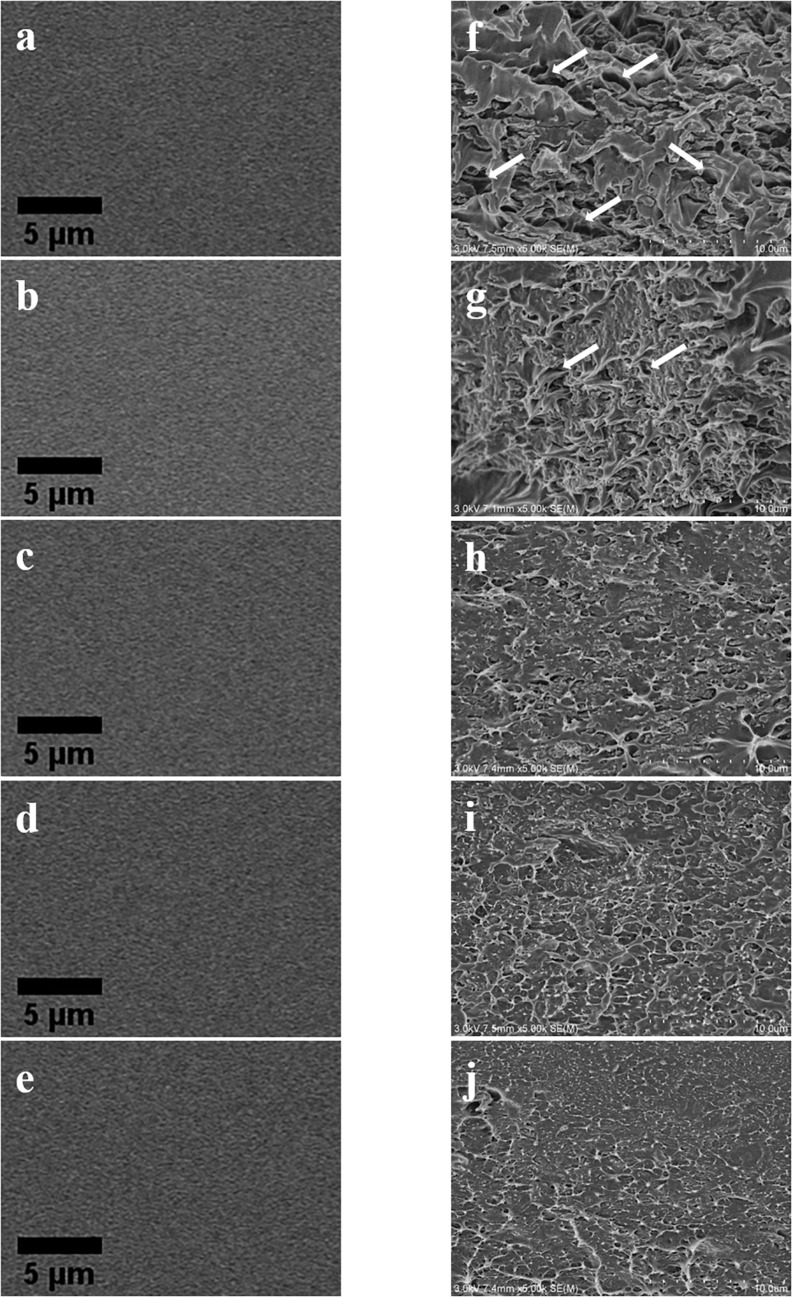
SEM images of surface and cross section of composite films. **(a)**∼**(e)** Surface; **(f)**∼**(j)** cross section. Amount of ASCNC: **(a)** and **(f)** 0%; **(b)** and **(g)** 2%; **(c)** and **(h)** 6%; **(d)** and **(i)** 9%; **(e)** and **(j)** 12%.

As shown in the SEM image (Figures 8a–e), there were no significant changes in the surface of the composite films whether or not ASCNC was added to the films. On the contrary, the cross section of the composite films was significantly different based on liquid nitrogen brittle fracture. There were many voids in the cross section of the ASH/PVA film (Figure 8f, white arrow), and the structure was loose. In contrast, the cross section of the composite films (Figures 8g–j) appeared densely compact with fewer voids by added ASCNC, and this phenomenon was accompanied by an increase in the amount of cellulose added. These results indicated that ASCNC could be uniformly dispersed and fully bonded in the ASH/PVA matrix (Fortunati et al., 2013). When the ASCNC loading exceeds 9%, the voids in the cross section of the composite films become almost invisible, which might be the reason for the improvement of the mechanical properties and the performance of the water barrier.

## Conclusion

*Artemisia selengensis* straw is rich in lignocellulose and is a promising biomass resource for biofilm materials. In this study, ASH and ASCNC of the lignocellulose components in *A. selengensis* straw were separately extracted. An ASCNC-enhanced ASH/PVA film was prepared by adding ASCNC to PVA and ASH substrates, indicating improved mechanical properties. The tensile strength of the composite film was increased by 80.1% to 36.2 MPa by adding 9% ASCNC. The water barrier of the composite film has been enhanced with ASCNC, with a lower WVP of 15.23%, by adding 12% ASCNC. Separation of the lignocellulosic components of *A. selengensis* straw and thereafter using them in the production of bio-composite films with improved properties contributes to reusing and reducing agricultural wastes.

## Data Availability Statement

All datasets generated for this study are included in the article/supplementary material.

## Author Contributions

All authors listed have made a substantial, direct and intellectual contribution to the work, and approved it for publication.

## Conflict of Interest

The authors declare that the research was conducted in the absence of any commercial or financial relationships that could be construed as a potential conflict of interest.
